# Continuous Rod Load Monitoring to Assess Spinal Fusion Status–Pilot In Vivo Data in Sheep

**DOI:** 10.3390/medicina58070899

**Published:** 2022-07-06

**Authors:** Markus Windolf, Maximilian Heumann, Viktor Varjas, Caroline Constant, Manuela Ernst, Robert Geoff Richards, Hans-Joachim Wilke, Lorin Michael Benneker

**Affiliations:** 1 AO Research Institute Davos, 7270 Davos, Switzerland; markus.windolf@aofoundation.org (M.W.); viktor.varjas@aofoundation.org (V.V.); caroline.constant@aofoundation.org (C.C.); manuela.ernst@aofoundation.org (M.E.); geoff.richards@aofoundation.org (R.G.R.); 2 Institute of Orthopaedic Research and Biomechanics, Trauma Research Center Ulm, Ulm University, 89081 Ulm, Germany; hans-joachim.wilke@uni-ulm.de; 3 Spine Unit, Sonnenhof Spital, 3006 Bern, Switzerland; lorinbenneker@sonnenhof.ch

**Keywords:** spinal fusion, implant load monitoring, implantable sensor, continuous measurement

## Abstract

*Background and Objectives*: Spinal fusion is an effective and widely accepted intervention. However, complications such as non-unions and hardware failures are frequently observed. Radiologic imaging and physical examination are still the gold standards in the assessment of spinal fusion, despite multiple limitations including radiation exposure and subjective image interpretation. Furthermore, current diagnostic methods only allow fusion assessment at certain time points and require the patient’s presence at the hospital or medical practice. A recently introduced implantable sensor system for continuous and wireless implant load monitoring in trauma applications carries the potential to overcome these drawbacks, but transferability of the principle to the spine has not been demonstrated yet. *Materials and Methods*: The existing trauma sensor was modified for attachment to a standard pedicle-screw-rod system. Two lumbar segments (L2 to L4) of one Swiss white alpine sheep were asymmetrically instrumented. After facetectomy, three sensors were attached to the rods between each screw pair and activated for measurement. The sheep was euthanized 16 weeks postoperatively. After radiological assessment the spine was explanted and loaded in flexion-extension to determine the range of motion of the spinal segments. Sensor data were compared with mechanical test results and radiologic findings. *Results*: The sensors measured physiological rod loading autonomously over the observation period and delivered the data daily to bonded smartphones. At euthanasia the relative rod load dropped to 67% of the respective maximum value for the L23 segment and to 30% for the L34 segment. In agreement, the total range of motion of both operated segments was lower compared to an intact reference segment (L23: 0.57°; L34: 0.49°; intact L45: 4.17°). Radiologic assessment revealed fusion mass in the facet joint gaps and bilateral bridging bone around the joints at both operated segments. *Conclusions*: Observations of this single-case study confirm the basic ability of continuous rod load measurement to resolve the spinal fusion process as indicated by a declining rod load with progressing bone fusion. A strong clinical potential of such technology is eminent, but further data must be collected for final proof of principle.

## 1. Introduction

Spinal fusion by arthrodesis of one or more spinal segments is a common intervention after instability, degenerated intervertebral discs (IVD), or scoliosis, among others [1]. However, the reported success rates of fusion procedures vary widely, with an incidence of failed bony union ranging from 5% to 35% in the lumbar spine [2]. In addition, asymptomatic patients with pseudoarthrosis often remain undetected [3,4]. Failed bony union can result in overloaded instrumentation leading to hardware failure as another frequent complication after spinal fusion surgery [5]. Arthrodesis of the spine is typically assessed by various imaging techniques, such as X-rays, computed tomography (CT) or magnetic resonance imaging. Interpretation of the imaging data is highly subjective, with inherent difficulties for diagnosing the state of union [6,7]. These issues are further highlighted by several studies showing a discrepancy between the assessment of fusion by radiologic examination compared with evaluation by surgical exploration [4,6,7], which still remains the gold standard for diagnosing a spinal non-union [3,4]. Furthermore, image-based diagnostic methods allow assessment only at distinct timepoints, requiring the presence of the patient in the hospital or practice. These shortcomings of current aftercare procedures lead to delayed diagnosis of healing complications (>six months), where a fast response is desired.

Implantable sensors generating in vivo data are of interest in trauma and spinal research [8,9]. Such devices may provide objective measures to enhance postoperative patient care. For example, quantifiable in vivo data may facilitate the assessment of spinal fusion progress. Treatment therapies could be tailored to the patient’s needs in real-time to foster bone consolidation and promote early mobilization. At the same time, follow-up visits, and consequently radiation exposure, could be kept to a minimum by radiation free remote monitoring, reducing the potential risk of neoplasia. However, current approaches to implantable sensors for the spine are considered premature. They face technical challenges, such as the selection of an appropriate sensing modality or power management issues [8]. One approach in spinal application is the measurement of implant loading by strain sensing in combination with posterior instrumentation (pedicle-screw-rod system) [10,11,12,13]. The load carried by the instrumentation creates measurable strain on the rod surface. As spinal fusion progresses, the initial implant load is gradually taken over by the fusion mass, unloading the instrumentation and decreasing the strain on the rod [14]. This principle has been investigated in the spine since 1966 by taking isolated measurements from time to time over the fusion period [15]. However, proving the measurement principle in vivo appears challenging. While Szivek et al. [16] observed a decrease in bone strain during fusion in a single patient, Rohlmann et al. [17] could not demonstrate a distinct response of fixator loading on the fusion process in ten patients with an instrumented screw-rod system. As opposed to these passively powered snapshot measurements, a novel approach involving the continuous measurement of implant loading has been recently developed by our group for trauma applications [9,18,19,20]. The system comprises an implantable datalogger which can be releasably attached to conventional bone plates. The sensor autonomously collects implant load data in a continuous fashion as an explicit metric to access bone healing. Wireless data synchronization via the patient’s smartphone allows for remote monitoring by the treating physician. While the trauma system is currently maturing into a clinically applicable device, the question is raised whether the underlying principle can be translated to other clinical applications such as spinal fusion.

Hence, the purpose of this study was to investigate the transferability of the continuous implant load measuring principle to the spine for determining the progress of spinal fusion. Preliminary in vivo data from a modified sensor system applied to one sheep is presented in the following.

## 2. Materials and Methods

### 2.1. Implantable Sensor System

An existing implantable sensor prototype, designed for plate load measurement in long bone fractures [20], was used in this study in a modified form. The sensor is actively powered by a battery, made for implant applications, to operate continuously and autonomously. A set of two resistive strain gauges configured in a Wheatstone half-bridge are glued to the sensor’s biocompatible and hermetic titanium enclosure between both fixation holes for screw attachment of the device to a standard locking plate. The strain gauges are oriented in a way to predominantly pick up longitudinal bending and axial loading of the implant. To minimize energy consumption, the device is activated by patient movement by means of an onboard accelerometer. The raw strain signal is amplified, sampled at 10 Hz and processed in real-time by an integrated microprocessor. A loading event is defined as the amplitude between a minimum and maximum in the signal progression exceeding a predefined threshold followed by a subsequent drop of the signal below a predefined level. All loading events throughout a day (0–24 h) are counted. The 50 highest loading events are retrieved from the data set and averaged to derive “implant load” (IL). Such statistical parameters are permanently stored in the internal memory of the datalogger and transmitted wirelessly and autonomously once per day via Bluetooth low energy to a bonded smartphone with a custom-made software application. To reduce data volume and energy consumption, raw data is neither saved on the datalogger nor transmitted. The statistical parameters are uploaded to a cloud server allowing remote monitoring of the implant load progression. A seven-day moving average filter (trailing) is applied to the implant load data to reduce scattering. To derive relative implant load (RIL), the IL data is normalized to the maximum IL recorded over time. Another computed outcome parameter is the daily activity time of the patient in percent, derived from the time of accelerometer activation per day.

**Figure 1:**
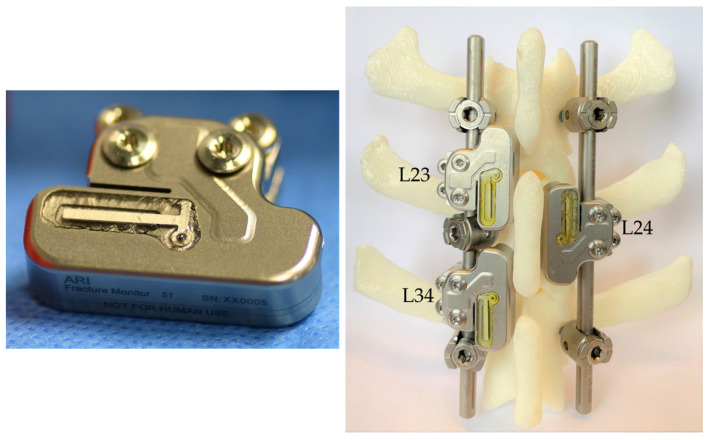
(**Left**) Spine sensor prototype as modified from an existing trauma sensor. (**Right**) Posterior instrumentation arrangement with attached sensors as used in the in vivo study on a 3D-printed ovine spine model (L2–L4).

For spinal application, the enclosure of the existing trauma sensor was trimmed to allow attachment to a standard screw-rod system in between the pedicle screws at a lumbar sheep spine (Figure 1). Two customized stainless-steel clamps secured the sensor to a 5.5 mm rod. The electronics, including strain gauge arrangement and microprocessor firmware, remained unchanged, despite adjustment of the accelerometer configuration to account for a different movement magnitude at the spine as compared to limb motion.

Before entering into in vivo experiments, basic functionality of the modified sensor system was verified in terms of signal sensitivity, linearity and cyclic signal stability on an instrumented plastic spine model, loaded on a material testing machine in flexion-extension. Surgical feasibility was confirmed in a wet-lab setting on a sheep cadaver.

### 2.2. Animal Study

After obtaining permission of the local ethics committee (Canton of Grisons, Switzerland, approval: TVB 202010), the animal study was performed in our facility accredited by AAALAC. One Swiss white alpine sheep (three years old, 80 kg weight) underwent posterior spinal fusion surgery in two consecutive lumbar motion segments (L23 and L34) to achieve facet joint arthrodesis under general anesthesia. Prior to anesthesia, the sheep was maintained off-feed for 48 h. The sheep was sedated with Detomidine (0.04 mg/kg intramuscular (IM)) before anesthesia induction using an intravenous mixture of Midazolam (0.2 mg/kg intravenous (IV)) and Ketamine (4 mg/kg IV). Afterwards, the sheep was endotracheally intubated and maintained under general anesthesia using Sevoflurane volatile liquid anesthetic (2–4% *v*/*v* in 0.5 L/min oxygen and air). Analgesia was provided preoperatively by a non-steroidal anti-inflammatory drug (Carprofen, 1.4 mg/kg IV) and lumbosacral epidural analgesia with Buprenorphine (0.005 mg/kg added to 0.9% saline for a total 10 mL volume). The antibiotic prophylaxis regimen included perioperative sodium-ceftiofur (2.2 mg/kg IV preoperatively and repeated every 90 min) followed by long-acting ceftiofur administered at the end of surgery (6.6 mg/kg IM).

**Figure 2:**
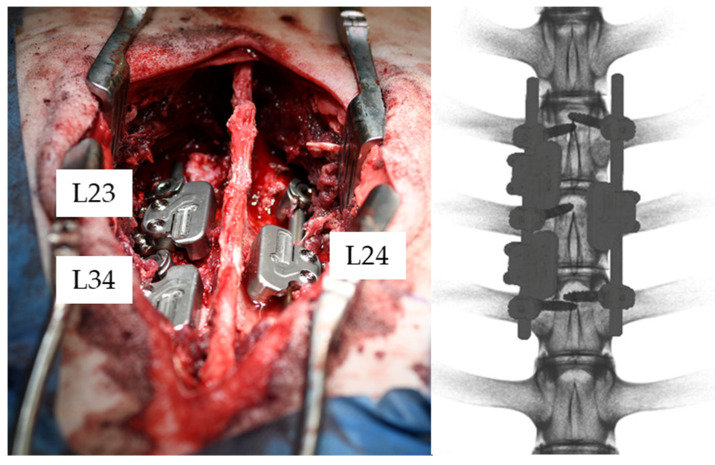
(**Left**) Intraoperative image after spinal instrumentation and placement of the sensors (L23, L34, L24). (**Right**) Posterior-anterior view from the postoperative CT scan (L1–L5).

The surgery was performed by an experienced spine surgeon together with a veterinarian surgeon. The surgical procedure comprised a bilateral excision of the facet joints with subsequent bi-segmental posterior instrumentation from L2 to L4 using a standard pedicle-screw-rod system (EXPEDIUM 5.5 Spine System, DepuySynthes Inc., Raynham, MA, USA). On the left side of the spine, pedicle screws (REF: 179712530, DepuySynthes Inc., Raynham, MA, USA) were inserted in all three vertebrae, whereas on the contralateral side the middle vertebra (L3) was bridged (Figure 1 and Figure 2). An asymmetric instrumentation was used to mimic different fixation configurations commonly used in human spinal fusion. No additional bone graft was placed either on the excised facet joints or between the transverse processes. The sensor clamps were attached to precut straight rods (REF: 179762120, DepuySynthes Inc., Raynham, MA, USA). The rods were then inserted in the heads of the pedicle screws.

The sensors were checked intraoperatively to ensure functionality before implantation. One sensor was attached between each screw pair with the electronic housing facing to the medial side of the rod (Sensor: L23, L34, L24; Figure 2). The sensors were rotated to arrange the upper surface as parallel as possible to the sheep’s frontal plane. Therefore, the measured loading mode is predominantly flexion-extension, as the rods are mainly subjected to bending [14]. After implantation, each datalogger was bonded with a smartphone and the data acquisition was started.

The sheep was able to fully weight-bear immediately after surgery. Postoperative analgesia consisted of repeated non-steroidal anti-inflammatory treatments for five days (Carprofen, 4 mg/kg subcutaneous every 48 h), Buprenorphine (0.05 mg/kg IM at the end of the surgical procedure, and every 6–8 h for 12–18 h) and transdermal Fentanyl (2 μg/kg/h transdermal patch for 72 h, applied at the time of surgery). An animal welfare assessment was performed and recorded using a score sheet twice daily for the first 3 days postoperatively followed by daily evaluation for four additional days, and then once weekly. The sheep’s weight was monitored weekly. A clinical CT (Revolution Evo, GE Healthcare Chicago, IL, USA) scan was performed in a four-week interval until euthanasia. An assessment of the clinical CT scan was conducted after eight weeks by five expert spine surgeons to replicate current clinical practice in determining fusion status and retrospectively compare the findings with the corresponding sensor outcome. At the time of the assessment the experts had not seen the sensor data to avoid bias.

### 2.3. Postmortem Evaluation

The sheep was euthanized 16 weeks postoperatively by means of an intravenous overdose of Pentobarbital (7.5 g IV) and the sensor measurement was stopped. The lumbar spine from vertebrae L2 to L5 was explanted and visually inspected. The non-operated L45 segment served as intact reference for mechanical testing. Postoperative and postmortem clinical CT scans were segmented and registered to each other using Amira3D software (Amira3D 2021.1, Thermo Fisher Scientific, Waltham, MA, USA) to screen for instrumentation or sensor loosening. After removal of instrumentation and sensor, a high-resolution peripheral quantitative CT scan (HR-pQCT, XtremeCT, Scanco Medical AG, Brüttisellen, Switzerland) was performed to assess the state of fusion. Solid fusion was defined as the absence of joint space between adjacent facet joint surfaces.

**Figure 3:**
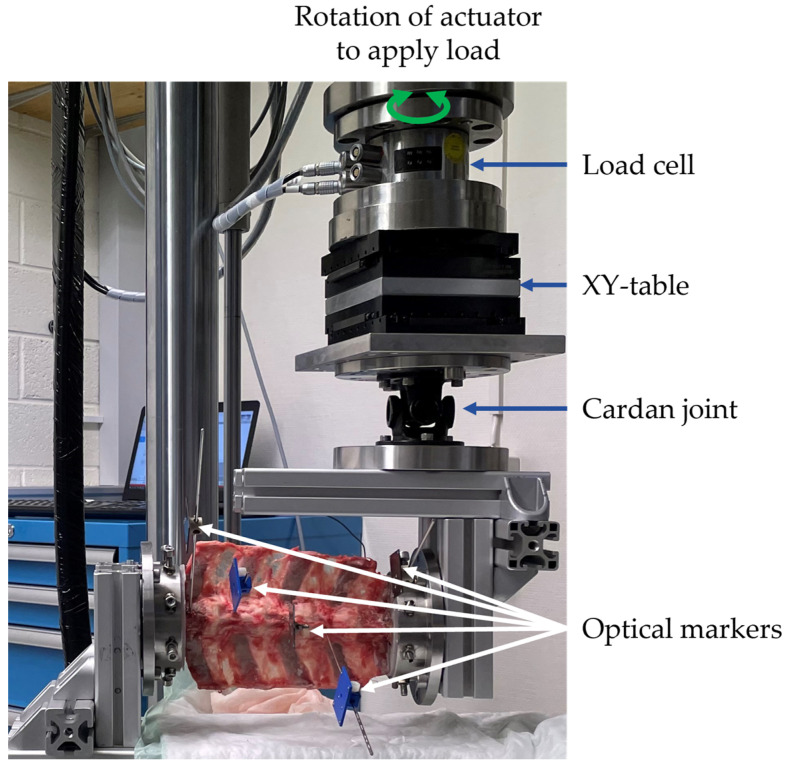
Test setup for unconstrained flexion-extension loading.

The spine was stored at −20 °C until mechanical testing for load-deformation behavior of the individual spinal segments under flexion-extension loading. After thawing, the specimen was kept moist throughout the experiment with Ringer’s solution. Vertebrae L2 and L5 were embedded in Polymethylmethacrylate (PMMA, SCS-Beracryl, Suter Kunststoffe AG, Fraubrunnen, Switzerland) and mounted on a customized test setup on an universal testing machine (MTS 858 Mini Bionix II, MTS Systems, Eden Prairie, MN, USA; Figure 3). The test setup allowed unconstrained movement in five degrees of freedom of the spine: anterior-posterior and cranial-caudal translation, lateral shear, lateral bending, and axial rotation. A flexion-extension moment was applied to the specimen in a quasistatic manner in angle control at 1°/s until ± 7.5 Nm was reached [21,22]. Optical marker-sets were fixed to the anterior aspects of the vertebral bodies. The three-dimensional motion of each vertebra in relation to the adjacent vertebra was tracked with an optical motion capture system (Aramis SRX, GOM GmbH, Braunschweig, Germany; Figure 3). Three test cycles were run and the mid of the second to the mid of the third cycle were evaluated [23]. Range of motion (ROM) and neutral zone (NZ) of each motion segment under flexion-extension were computed [24,25]. ROM is defined as the difference in motion between the neutral position and the deflection at maximum moment for flexion and extension separately. NZ is the portion of the ROM where the spine can move freely without load being applied.

Subsequently, a cyclic mechanical test was performed to investigate the stability of the sensor signal in the implanted configuration. The spine was reinstrumented, equipped with sensors and mounted on the test machine. Sinusoidal flexion-extension loading with an amplitude of ±6 Nm was applied at a frequency of 0.25 Hz for 42,000 cycles. The loading magnitude of 6 Nm was chosen based on the average sensor signal amplitude as measured at the end of the study (16 weeks). During the test the specimen was kept moist with gauzes wrapped around the spine, soaked with Ringer’s solution. Additionally a plastic foil was covering the wet gauzes to prevent them from drying out [22]. At the beginning of the test, the sensor signal was closely monitored along with the motion data in increments of 600 cycles. Towards the end of the cyclic test monitoring took place at cycle 33,600, 37,800, and 42,000. To investigate the relative signal change, the first sensor measurement was set to 100% and the following measurements were plotted as a fraction of it.

## 3. Results

Throughout the study the sheep remained healthy and active and tolerated the procedure and implanted hardware well. The sensors functioned reliably and synchronized all collected data with the smartphones.

**Figure 4:**
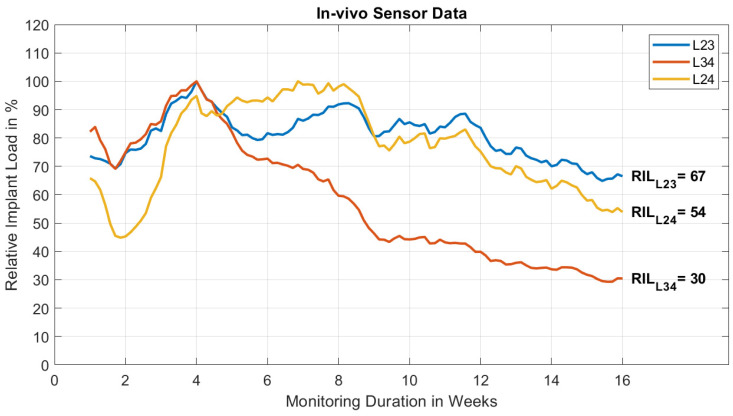
Recorded relative implant load (RIL) over time for the three sensors.

The daily activity time and number of loading events as detected by the sensors decreased in the first two weeks and then stabilized on a constant level with minor fluctuations throughout the rest of the experiment. The average loading events recorded per day for sensors L23, L34, and L24 were 1376, 1277, and 922, respectively. Figure 4 shows relative implant load (RIL) measured by each sensor over time. From the second week RIL increased for all three sensors until reaching the maximum at week four for sensors L23 and L34 and at week eight for sensor L24. Afterwards, sensor L34 decreased constantly to an RIL of 30% at 16 weeks postoperatively. The more cranial sensor (L23) showed a reduction of implant load to 67%, after another increase in RIL of ~12% from week six to eight. The sensor bridging the two operated segments on the contralateral side showed a similar trend as sensor L23 to a final RIL of 54%.

Video S1 shows the raw signal of one sensor measured during walking of the sheep. A signal peak becomes apparent when the sheep was changing walking direction and subsequently turned its head.

**Figure 5:**
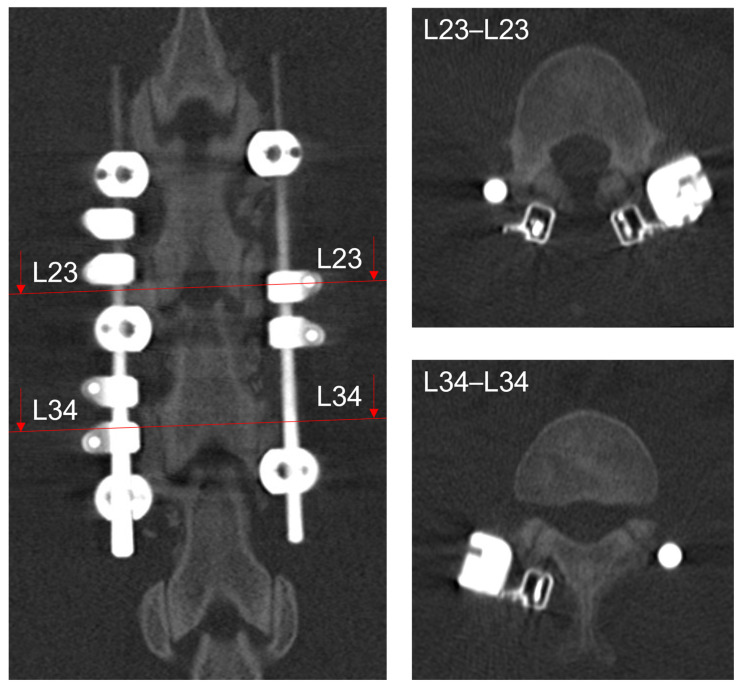
Sections of the eight-week clinical CT scan. Posterior-anterior view of operated spine (**left**) with section lines for cranial-caudal slices of the operated segments L23 and L34 illustrated on the **right**.

The CT at eight-weeks postoperative was judged by the five medical experts in a group discussion. After initial differences in perception the group reached the following verdict: at segment L23 onset of bone formation was declared, whereas the more caudal segment L34 was deemed to show a more mature fusion state referred to as the early state of fusion, with already bilateral bridging bone around the facet joints and the start of bone consolidation in the joint gap (Figure 5).

**Figure 6:**
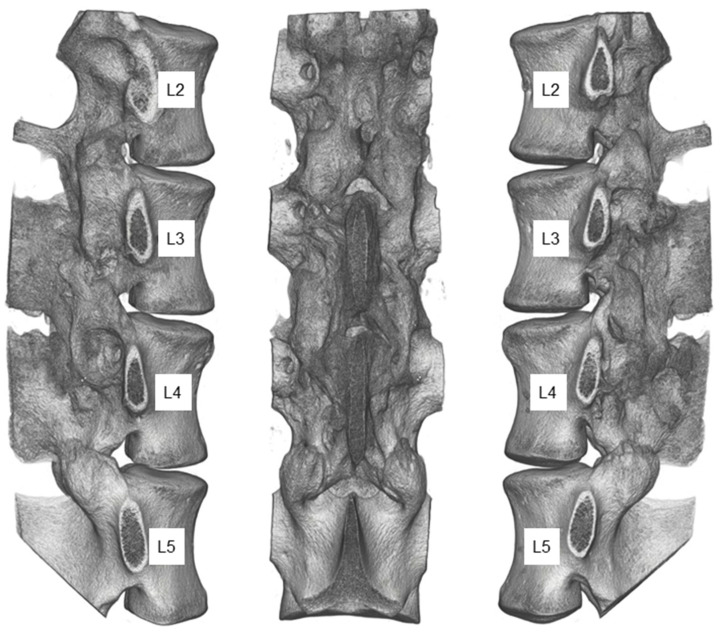
The lumbar spine’s lateral and posterior views (L2–L5) after euthanasia and implant removal derived from high-resolution CT data.

Registration of the postoperative and euthanasia CT scans revealed no changes in vertebra and instrumentation alignment. The HR-pQCT scan after euthanasia showed that the remaining facet joint surfaces of level L34 were solidified, with additional bilateral bridging bone around the joints (Figure 6). The more cranial segment had slightly less fusion mass in the joint space, but also bilateral bridging bone. Based on the radiological assessment, the control segment L45 appeared unimpaired by the performed procedure, with no signs of osteophytic growth between the vertebrae.

**Figure 7:**
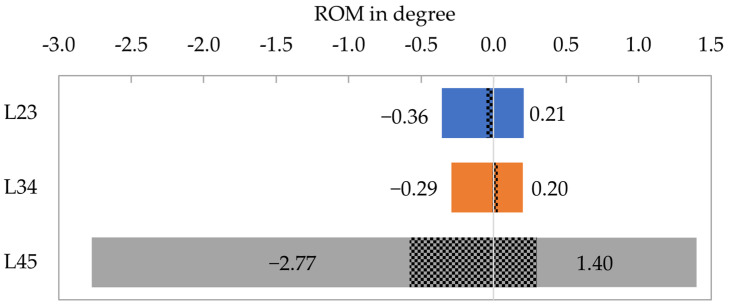
Range of motion (ROM) of the three spinal segments under 7.5 Nm flexion-extension loading. Positive values: flexion. Negative values: extension. The hatched area represents the neutral zone (NZ). L45: untreated control.

ROM after 16 weeks under 7.5 Nm flexion-extension loading is shown in Figure 7 for each segment. The operated segments exhibited a ROM of less than 0.4° in both loading directions. The total ROM (flexion + extension) of the two operated segments was similar (L23: 0.57°, L34: 0.49°). In contrast, the untreated control segment (L45) rotated 1.40° in flexion and −2.77° in extension. The NZ (hatched area in Figure 7) was small for both operated segments (<0.05°). In the intact segment L45 the NZ accounts for approximately 21% of the ROM in flexion as well as in extension.

**Figure 8:**
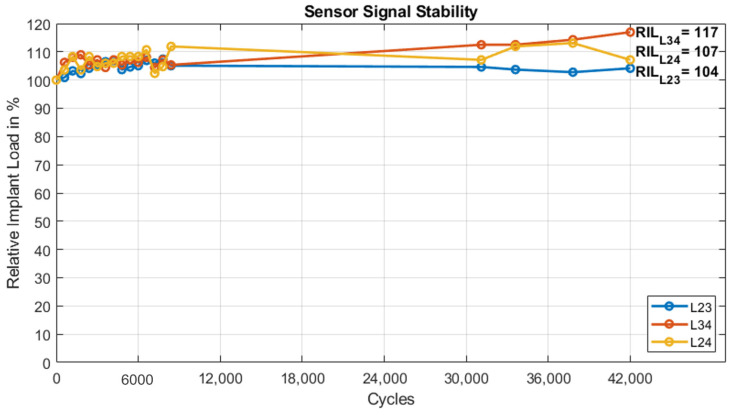
Relative implant load (RIL) during in vitro flexion-extension loading over 42,000 cycles for all sensors. Circles mark the data acquisition points.

Over 42,000 cycles of flexion-extension loading, the amplitude of the sensors L23, L34 and L24 increased by 4%, 17% and 7%, respectively (Figure 8). The total ROM of segment L23 increased by approx. 6%, whereas segment L34 increased by 37% over the course of testing. However, in absolute terms the total ROM remained small (<0.7°).

## 4. Discussion

The principle of continuous implant load monitoring has recently proven its potential as a relevant metric for assessing bone consolidation processes [20]. After having obtained encouraging results for long-bone healing, the question about transferability of the concept to spinal fusion is apparent. A sensor system, developed in our group for trauma applications, was therefore modified for rod load measurement and was applied in vivo in an ovine model with two-level lumbar instrumentation in one animal to obtain preliminary data to inform future developments. Obviously, this single-case study can only introduce the principle and suggest its basic feasibility without allowing resilient conclusions.

In our animal we observed mature posterolateral fusion (PLF) of the operated motion segments on CT after 16 weeks as confirmed by postoperative mechanical testing on range of motion (ROM). The fused segments were almost motionless with minor laxity in flexion-extension. For sensor data collection we utilized three redundant sensors in-between each pedicle screw-pair to maximize the data output on this single observation. In agreement with CT and the mechanical test, all sensors showed decreased rod load at the end of the study. This indicates that load was shifted from the instrumentation to the spine as a result of progressed fusion in both operated segments. Additional cyclic mechanical testing was performed to ensure the signal stability of the measurement-construct including sensor, instrumentation and spine to rule out settling, component loosening or sensor wear-out effects, which could otherwise explain the signal drop. On the contrary, we saw a moderate increase in the measured amplitude during cyclic testing which was in line with an increased ROM of the considerably stressed spinal cadaveric specimen. Therefore, we confidently conclude that the decrease in rod load can be attributed to stiffening of the operated motion segments due to fusion, confirming the basic measurement principle.

The feedback from five expert surgeons on the CT data after week eight was conclusive but also underlined the known difficulties of drawing objective judgements from medical image data with regard to spinal fusion status [4,6,7].

The utilized metric to access fusion, relative implant load (RIL), was defined as the average of the highest 50 loading events over one day. This approach, taken from the trauma application [20], intends to maximize the observable difference between non-fused and fused states. While the vast majority of loading events occur in the low-amplitude range, the higher magnitude events were found to be most predictive for resolving bony consolidation and, thus, are collected in a peak event buffer. However, this concept as used in trauma needs validation for the spine.

The difference in output of sensors L23 and L34 (67% vs. 30% RIL at 16 weeks) would intuitively suggest different fusion states. However, from postmortem testing, the ROM of both fused levels was similar. This can be the result of various reasons, such as different individual sensor sensitivities, different sensor to rod attachment strengths or orientations, varying pedicle screw anchorage in the vertebrae or differences in force transmission through the screw-rod connections. Most important might be a potential crosstalk between both levels being spanned with a single instrumentation. An influence of one fusion level on the adjacent sensor cannot be excluded and is likely to occur. This, on the one hand, makes it more difficult to interpret a particular sensor signal, but on the other hand carries potential to measure multiple level fusion simultaneously. The asymmetric instrumentation was chosen to collect first data on such fusion crosstalk. The RIL curve of sensor L24 qualitatively resembles the outputs of both sensors on the contralateral side showing a final RIL of 54%. This falls in between the values of the two individual segments (67% and 30%) and indicates the possibility of monitoring multiple levels with a single sensor. However, the current results did not demonstrate that a bridging sensor signal may distinguish, for example, a fusing from an adjacent non-fusing segment. The relevance of RIL curves is currently not fully understood and requires in-depth investigation before a clear relation between relative implant load measurement and stability of a fused motion segment can be established.

Kanayama et al. demonstrated a maturation of fusion in an ovine model with additional autologous bone grafting at the eighth week based on X-ray findings, and proved that the spine was already biomechanically stable at this point [14]. In the same study, it was shown that rod strain decreased when PLF was progressing. In our study, a significant decrease in rod load could be seen only for sensor L34 at week eight. A direct comparison between the studies is not feasible due to differences in the used model and our single-case data. It is worth noting that, in long-bone trauma applications, the implant load signal of the sensor appears particularly sensitive to early changes in fracture stiffness [20], while in this first spinal fusion case the signal response seems to be less pronounced early on. Over 16 weeks, RIL dropped to 30% for sensor L34 and only to 67% for sensor L23, indicating that in the least sensitive configuration the fusion process was resolved by ~30% of implant load drop. However, unlike the typical curve progression in the trauma application, where implant load reaches a plateau at around 10–20% [20], rod load has not reached such equilibrium after 16 weeks and is likely to continue falling. This, hence, suggests that rod load measurement might be more sensitive for resolving late-stage fusion processes. A persisting residual signal can also relate to contractions of the spinal muscles passing over the sensor housing. The extent on influencing the measurement is unknown [26].

It needs to be stressed that the sensor design was not optimized for this application in terms of strain gauge orientation. Due to the axial alignment of the strain gauges, flexion-extension loading is deemed to have a predominant influence on the sensor signal, but it is expected that other loading conditions (lateral bending, axial rotation) also made an impact on the sensor signal. Superimposed loading at the rod is combined and transferred into a one-dimensional signal in the digital domain. The literature states that the largest relative difference in ROM between a bilaterally facetectomized and an intact motion segment can be measured in axial rotation [27,28]. Therefore, it is reasonable to assume that this relative difference can also be seen between facetectomized and fused states. Consequently, the measurement of torsion at the rod could deliver the clearest difference over the fusion process. Such questions must, however, be systematically investigated to inform future sensor designs.

Further limitations became apparent. The non-optimized shape of the sensor prototype, originally designed for a different use, made positioning on the posterior instrumentation challenging. With regard to human application, the sensor dimensions should be miniaturized to allow attachment of the sensor in the limited space at the human lumbar region and potentially enable minimal invasive insertion.

Another limitation concerned the selected in vivo model. While the qualitative ROM between the ovine and human spine is similar, values for the absolute ROM of the human spine vary widely in the literature and differ substantially from the ovine spine [29]. Furthermore, both anatomies, in particular the facet joints, exhibit notable differences [30]. Therefore, results are not directly transferable to the human situation. However, the ovine model is still considered most suitable for lumbar spinal fusion studies [29,31]. Ovine bone healing and bone remodeling activities are comparable to those in humans [32]. The sheep’s spine as a quadruped is mainly exposed to axial loading, similar to humans with even slightly higher axial load values [33]. Smit et al. suspected that a higher stress in the sheep spine is decisive for the increased density of the trabecular bone. It is, furthermore, expected that due to this increased bone density, hardware in the spine experiences better mechanical hold. In addition, the higher mechanical stimuli might be attributed to a better fusion rate in quadrupeds than in humans [33]. Therefore, care must be taken to compare the fusion status in this ovine study after 16 weeks with the fusion process in clinics.

Szivek et al. investigated the changes of bone strain on the lamina of thoracic vertebrae and strain on the stabilizing hardware in a single patient to determine the onset of fusion [16]. They showed that in specific loading modes of the patient it can be possible to measure changes on bone strain when fusion is progressing. Strain measured on the rod also decreased over time, but this was partially a result of strain gauge detachment from the hardware. In another study investigating strain changes on implants during spinal fusion, Rohlmann et al. used an instrumented telemetric fixator to perform snapshot rod deflection measurements on ten patients. The measured loads on the implant during different patient activities did not indicate a distinct reduction of implant load over the spinal fusion process. They concluded from their clinical data that assessing fusion through rod load measurement is difficult [17]. We hypothesized that continuous monitoring of rod deflection with statistical data processing could overcome those difficulties and detect fusion by averaging out variances in functional loading. Results from our preliminary study support this hypothesis. The system could be used in the future in a research context to compare implants, medications or treatment strategies in preclinical [34] or clinical settings, and could allow the assessment of study groups based on quantitative in vivo data. The question remains whether such a monitoring system carries clinical relevance beyond being a useful research tool. Instrumented posterolateral fusion (PLF) amounts to more than a third of all adult lumbar fusion procedures for degenerative disorders [35]. The fusion rate for this particular technique is reported at 84.9%, but the clinical success rate is only 40.4% [35]. In this domain, PLF has a lower success rate than interbody fusion [2]. From a clinical perspective it would be desirable to detect fusion occurrence early on, and react to healing disturbances or hardware failures, which mainly occur in the first six months after surgery [36]. Based on quantifiable data provided by the sensor, a personalized rehabilitation protocol could be implemented, allowing the controlled steering of patient activity. Also, activity data e.g., from integrated inertial measurement units could play an important role in individualized after-care concepts. Here, the on-board accelerometers mainly served the purpose of reducing energy consumption. However, two important conclusions can be drawn from the acceleration data: (1) The pronounced decline in active time from post-operation to later stages suggests variable loading at the spine. This was reflected in the RIL curves, but did not obstruct the effect of fusion; and (2) the active time was similar between the sensors validating their function.

With further research on RIL in relation to the state of fusion, such sensor systems could help to determine the timepoint of a biomechanically stable spine targeting early removal of the instrumentation, which is of particular interest in young patients. Interbody fusion is another frequently performed procedure with a similar complication profile as PLF and is, hence, considered another target indication for such sensor systems. Transferability of the measurement principle to interbody fusion is unclear and must be investigated in the future.

Implant load characteristics in the spine are widely studied. However, most investigations are conducted on cadaveric specimens or in silico [11,14,37,38]. Only very little is known about in vivo loads and load changes during the fusion process [17,39]. To our knowledge, this study presents the first continuously measured in vivo dataset on posterior instrumentation loading after posterolateral fusion surgery.

## 5. Conclusions

An existing implantable sensor prototype was successfully transferred from trauma to spinal application. Observations of this single-case animal study confirmed the basic ability of continuous rod load measurement to resolve the spinal fusion process as indicated by declining sensor signals with progressing fusion. A strong clinical potential of such technology is eminent, but further data must be collected for final proof of principle.

## Data Availability

The data presented in this study are available on request from the corresponding author.

## Supplementary Materials

The following supporting video can be downloaded at: https://www.mdpi.com/article/10.3390/medicina58070899/s1, Video S1: Live stream of sensor signal recorded in vivo during walking and head movement of the sheep.
